# Advanced MXene-Based Micro- and Nanosystems for Targeted Drug Delivery in Cancer Therapy

**DOI:** 10.3390/mi13101773

**Published:** 2022-10-19

**Authors:** Fatemeh Mohajer, Ghodsi Mohammadi Ziarani, Alireza Badiei, Siavash Iravani, Rajender S. Varma

**Affiliations:** 1 Department of Organic Chemistry, Faculty of Chemistry, Alzahra University, Tehran 19938-93973, Iran; 2 School of Chemistry, College of Science, University of Tehran, Tehran 14176-14411, Iran; 3 Faculty of Pharmacy and Pharmaceutical Sciences, Isfahan University of Medical Sciences, Isfahan 81746-73461, Iran; 4 Regional Centre of Advanced Technologies and Materials, Czech Advanced Technology and Research Institute, Palacký University in Olomouc, Šlechtitelů 27, 783 71 Olomouc, Czech Republic

**Keywords:** MXene-based systems, nanocomposites, cancer nanotherapy, photothermal therapy, targeted drug delivery

## Abstract

MXenes with unique mechanical, optical, electronic, and thermal properties along with a specific large surface area for surface functionalization/modification, high electrical conductivity, magnetic properties, biocompatibility, and low toxicity have been explored as attractive candidates for the targeted delivery of drugs in cancer therapy. These two-dimensional materials have garnered much attention in the field of cancer therapy since they have shown suitable photothermal effects, biocompatibility, and luminescence properties. However, outstanding challenging issues regarding their pharmacokinetics, biosafety, targeting properties, optimized functionalization, synthesis/reaction conditions, and clinical translational studies still need to be addressed. Herein, recent advances and upcoming challenges in the design of advanced targeted drug delivery micro- and nanosystems in cancer therapy using MXenes have been discussed to motivate researchers to further investigate this field of science.

## 1. Introduction

Today, the development of nanotechnology has resulted in the creation of several multidisciplinary research domains, namely bionanotechnology, with special contributions in biomedicine and cancer nanomedicine [1,2,3,4,5,6]. An assortment of nanoparticles and nanoarchitectures has been designed with satisfactory biocompatibility and targeting properties for a wide range of biomedical and clinical applications; however, due to the stringent regulatory necessities, only a handful of them have entered into clinical trials/studies. Nevertheless, these materials have garnered widespread attention from scientists for their deployment as anticancer nanocarriers and nanobiosensors [7,8,9,10,11,12].

Chiral peptide/protein-derived supramolecules with biological activities can assist in prevention and treatment of various diseases, especially cancers [13]. Biomedicine entails ground-breaking supplies and technologies for specific therapeutics. Traditional policies are not in favor relative to modern medicine due to vital shortcomings, such as nonexact targeting, as well as the absence of synergistic attributes, wherein the booming development of nanotechnologies brings forth a fresh outlook to the biomedicine arena [14,15,16]. In this context, the design of nanomaterials with therapeutic and diagnostic potential has been widely noticed by researchers. For instance, two-dimensional (2D) hexagonal boron nitride (2D h-BN) flatlands have garnered much attention due to the optical and physicochemical properties, outstanding thermal/chemical constancy, and biocompatibility [17,18], showing excellent potential in biomedicine [19].

On the other hand, with the discovery of graphene [20], transition metal dichalcogenides (e.g., TiS_2_, MoS_2_, and WS_2_) [21,22,23], black phosphorus nanosheets [24], layered double hydroxides [25], graphitic carbon nitride [26], boron nitride [27], metal–organic frameworks [28], and transition metal oxides [29], among others, a variety of advanced micro- and nanosystems have been designed for cancer therapy. Recently, MXenes, 2D material comprising transition metal carbides, nitrides or carbonitrides, have captured widespread attention in photothermal therapy (PTT) in view of the high light-to-heat conversion efficiency, elevated paramagnetic performance, and large specific surface area [30,31,32]. However, MXene photothermal agents (PTAs) are unstable in aqueous/oxidative surroundings as their terminal functional groups (–F, –OH, or –O) are vulnerable to oxidation and tend to aggregate in physiological solutions; although, after suitable modification/functionalization, the stability and properties of these structures can be improved [33].

Among the recently introduced materials, MXenes are two-dimensional (2D) transition metal carbides and nitrides with a vast surface area and hydrophilicity, which have been explored in biomedical engineering and medicine [34,35,36]. They have swiftly become popular for a range of study domains due to their outstanding features since Yury Gogotsi and colleagues synthesized the first family member in 2011 [37]. Although MXenes are a relatively young family of 2D materials, the number of scientific articles connected to them nearly doubles every year [30,38,39,40,41].

**Figure 1:**
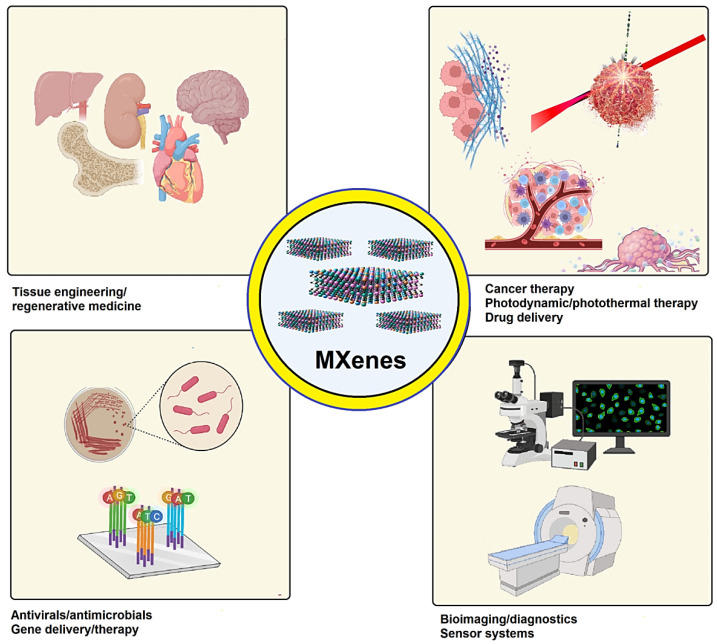
MXenes and their derivatives with versatile biomedical potentials.

Two-dimensional MXenes are contemporary 2D transition metal carbides, which resemble graphene sheets comprising the formula M_n+1_X_n_T_x_ (e.g., Ti_3_C_2_T_x_). Exfoliation of the “A” layer from a “MAX” predecessor—where M, A, and X indicate the transition metal, elements from IIIA or IVA, and N, respectively—can be used to make these materials, depending on their situation and type of applications. Among widely developed methods deployed for the manufacturing of MXenes comprise urea glass (e.g., Mo_2_C and Mo_2_N) [42], chemical vapor deposition (e.g., Mo_2_C) [43], molten salt etching (e.g., Ti_4_N_3_Tx) [44], hydrothermal fabrication (e.g., Ti_3_C_2_Tx) [45], and electrochemical synthesis (e.g., Ti_2_CTx). The wet etching method, in particular, may be utilized to create them in good quality with largely hydrophilic properties [46]. MXenes and their derivatives can be applied for photothermal and photodynamic cancer therapy [47]. These materials with unique optical properties have been explored in the field of (bio)imaging and (bio)sensing [48,49,50]. On the other hand, routinely applied anticancer drugs for chemotherapy may suffer from low specificity/selectivity, high toxicity, off-target toxic effects, and inappropriate clearance. Designing multifunctional systems with high biocompatibility, specificity/selectivity, biodistribution, and bioavailability can help to improve the efficiency of cancer therapy and tumor ablation. MXene-based micro- and nanosystems have been applied for targeted delivery of anticancer drugs/therapeutic agents in cancer therapy after surface functionalization/modification by bioactive/biocompatible agents (Figure 1) [51,52].

In addition, long-acting therapeutic anticancer drugs may cause possible toxic effects on normal cells along with a low efficacy in cancer therapy [53,54,55]. Low bioavailability of conventional chemotherapy, with some drawbacks, such as possible side effects, high dose requirements, and multidrug resistance, have prompted researchers to find new solutions based on drug delivery micro- and nanosystems with the benefits of higher solubility, specific targeting, high biocompatibility, selectivity, and reduced toxicity [56,57]. Conventional organics with biodegradable attributes and strong biocompatibility have been extensively studied for clinical applications, but their poor thermal and chemical stability and low functionality are some of the obstacles limiting their clinical developments [58]. In this context, MXene-based nanostructures have unveiled a variety of clinical and biomedical potential owing to their unique physicochemical properties, such as ease of functionalization, tunable structures, biosafety, and physiological constancy [59]. However, crucial aspects of toxicity and biocompatibility ought to be further explored. The potential toxicity of MXenes has been investigated throughout the embryonic and angiogenesis phases, wherein they have revealed negative impacts during the early stages of embryogenesis [58]; about 46% of MXene-exposed embryos died within 15 days [60]. MXenes were shown to impede the angiogenesis of the embryo’s chorioallantoic membrane after 5 days at the tested dosages. When compared to the controls, several genes were deregulated in the liver tissues and brain of embryos treated with MXene; nevertheless, further research and analysis are warranted to comprehend the potential toxicity of MXenes [60,61]. The biosafety of MXenes can be improved by applying suitable functionalization and hybridization techniques using biocompatible and biodegradable agents (e.g., cellulose and chitosan) along with optimized reaction/synthesis conditions.

The key applications of MXenes in biomedicine comprise, among others, (bio)imaging, tissue engineering, (bio)sensing, cancer therapy/diagnosis, and drug delivery [62,63]. Properties of MXenes can be improved by using suitable hybridization techniques, providing MXenes with improved thickness and compactness of the silane film, uniformity, good density, and fewer flaws [64,65]; wherever conductivity is essential for interaction with electromagnetic waves, interference shielding may exploit appropriate magnetic dipoles. Additionally, 2D MXenes ranging from metallic to semiconductor can detect adjustable conductivity; 2D MXenes with hydrophilicity ought to be manufactured using cost-effective methods [64]. Their layered structures, on the other hand, are likely to hinder the creation of gaps, which may change their magnetic and electric characteristics. As a result, unfavorable effects on the electromagnetic interference activity of composites made from MXenes are not unlikely. This significant disadvantage could be resolved by synergistic interactions amongst MXenes and conducting/magnetic materials [64,66].

Studies on these materials expose the need of controlling their physicochemical characteristics through surface charge engineering for novel biotechnological and biomedical applications [67]. For cancer therapy and tissue-engineering purposes, MXene-based micro- and nanosystems offer adjustable mechanical properties, excellent photothermal conversion efficiency, targeting competence, biocompatibility, selectivity, and regulated drug discharge [68]. For instance, composite hydrogels based on Ti_3_C_2_ MXene and cellulose have been engineered to respond quickly to near-infrared (NIR)-stimulated features. These manufactured hydrogels modified with doxorubicin (DOX) hydrochloride exhibited exceptional drug release acceleration and could be employed as nanoplatforms for intratumoral cancer therapy [68].

## 2. Advanced MXene-Based Micro- and Nanosystems

### 2.1. Structural, Chemical, and Electronic Properties of MXenes

MXenes have functional groups such as F, OH, and O after its exfoliation from the MAX phase where the O- and/or OH- terminated groups are the most stable, as F group will be substituted with OH groups on rinsing and storing in H_2_O [69]. Xie et al. stated that OH groups could be transformed into O terminations over high-temperature and/or metal adsorption procedures [69]. In addition, O-terminated MXene could decompose into bare MXene up on interaction with Mg, Ca, Al, or other metals [70]. Modeling is crucial in designing MXenes with novel structures [71]. Surface groups are located above the hollow sites among three near C atoms [72,73]. The precise configurations of surface groups depend on both, their species and the MXene’s constituent materials [74]. MXenes have many usage such as, electronic [75], dielectric [76], magnetic [77,78,79,80], elastic [81], thermoelectric [82], and optical properties [83] as has been documented by computational studies. Bare MXene, such as Ti_n+1_X_n_, are known to be metallic in behavior [83]. In terms of X atoms, titanium nitrides exhibit more metallic properties than titanium carbides, simply because the N atom possesses one more electron than the C atom [84]. In terms of the chemical properties of MXenes, Naguib et al. reported that Ti_3_C_2_T_x_ are oxidized in air, CO_2_, or pressurized water [85] wherein the oxidation results in the formation of anatase TiO_2_ nanocrystals embedded in amorphous carbon sheets (TiO_2_-C hybrid structure).

One of interesting topics in the progression of theranostic nanomedicines for in vivo clinical studies is the finding of association among the physicochemical nature of the designed structures and their connections with living organizations. Therefore, experimental studies are necessary to assess the productivity, toxic effects, and their physio-chemical properties. Comprehensive preclinical studies and the assessment of the biological interactions of MXenes and their complexes in in vivo systems could support to find their limits in the future [86].

MXenes are important due to their widespread applications as supercapacitors [87], in electromagnetic interference shielding [88], optical and light detection [89], and even in communication and biology [90]. The optical properties of MXenes have displayed the possibility of producing electrodes [90], photonic devices and plasmonic applications [91]. Furthermore, biocompatible MXene (Ti_3_C_2_)-based composites (MnOx/Ti_3_C_2_) were applied for cancer theranostics, nanoplatform for photothermal cancer/tumor nanotherapy by photoacoustic imaging [92].

### 2.2. MXene-Based Biomimetic Plasmonic Method

**Figure 2:**
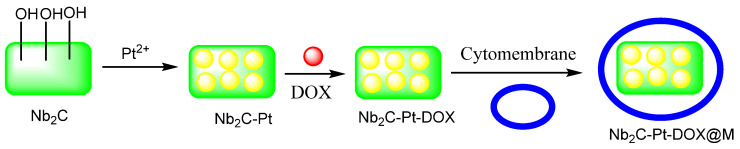
Schematic diagram of MXene-based biomimetic plasmonic assembly for targeted cancer therapy [94].

An effective plasmonic-enhanced technique has been presented and used for biomimetic photo-induced plasmonic assembly (NPD@M). Nb_2_C plasmon (MXene), Pt nanozymes, DOX, and tumor cytomembrane make up the assemblage for targeted cancer therapy. The hot-electrons created from MXene after homogeneous targeting and internalization into cancer cells notably promote the catalase-like and oxidase-like functions of Pt nanozymes to generate O_2_ and reactive oxygen species (ROS), combined with tumor-penetrating photothermal therapy. DOX is also released in acidic conditions, aided by O_2_ and ROS-induced suppression of P-glycoprotein (P-gp)-mediated drug efflux [93]. These results demonstrated that MXene-augmented nanozyme therapy decreased the HeLa cell viability by 38.67% when compared to control cells; nanozyme catalytic activity was improved by the hot-electron oscillations from Nb_2_C by NIR-II laser to kill tumors. NPD@M was inserted into the body by the tail vein so that it could enter the tumor through the EPR effect and tumor cytomembrane targeting while the NIR-II light illumination and photothermal possessions expanded tumor vessels to improve the blood pressure to a drug eruption. The nanozyme activity was boosted by the plasmon thermoplastic result. Using a high concentration of H_2_O_2_ high (100 μM~1 mM) in the tumor results in down-regulation of the hypoxia-inducible factor (HIF-1α). Concurrently, the heightened oxidase endorses ROS production which reduces the mitochondrial energy source to P-gp glycoprotein, while a membrane efflux pump recognizes the chemotherapeutic drugs for transporting out of cells. This process counters multidrug resistance and recovers the chemotherapy effectiveness. Collectively, biomimetic plasmonic assembly completes the tumor treatment by irradiation of NIR-II, as a novel strategy to promote the nanozyme biocatalyst and plasmonic application in tumors (Figure 2) [94].

### 2.3. MXene/DOXjade Platforms

For synergistic cancer therapy, Ti_3_C_2_-PVP@DOXjade, a pH-responsive dual-therapeutic compound based on deferasirox and DOX, was created wherein photo-irradiation with Ti_3_C_2_-PVP@DOXjade displayed a pH-responsive iron chelation/PTT/chemotherapy anticancer activity [95]. Cancer cells have a high requirement for iron with important roles in cell proliferation, tumor growth, and metastasis thus rendering iron metabolism a promising therapeutic target. Unfortunately, existing iron-based therapy techniques are frequently ineffective and have side effects. The prodrug combines deferasirox (ExJade^®^), a therapeutically authorized iron chelator, with DOX, a topoisomerase 2 inhibitor (DOX). DOXjade was loaded onto ultrathin 2D Ti_3_C_2_ MXene nanosheets to create Ti_3_C_2_-PVP@DOXjade, which allows DOXjade’s iron chelation and chemotherapeutic functions to be photo-activated at tumor sites, additionally potentiating a robust photothermal effect with photothermal conversion efficiencies up to 40%. Ti_3_C_2_-PVP@DOXjade promotes apoptotic cell death and down-regulates the iron depletion-induced iron transferrin receptor, according to antitumor mechanistic studies as iron chelation/photothermal/chemotherapy responds to the pH of the tumor [95].

**Figure 3:**
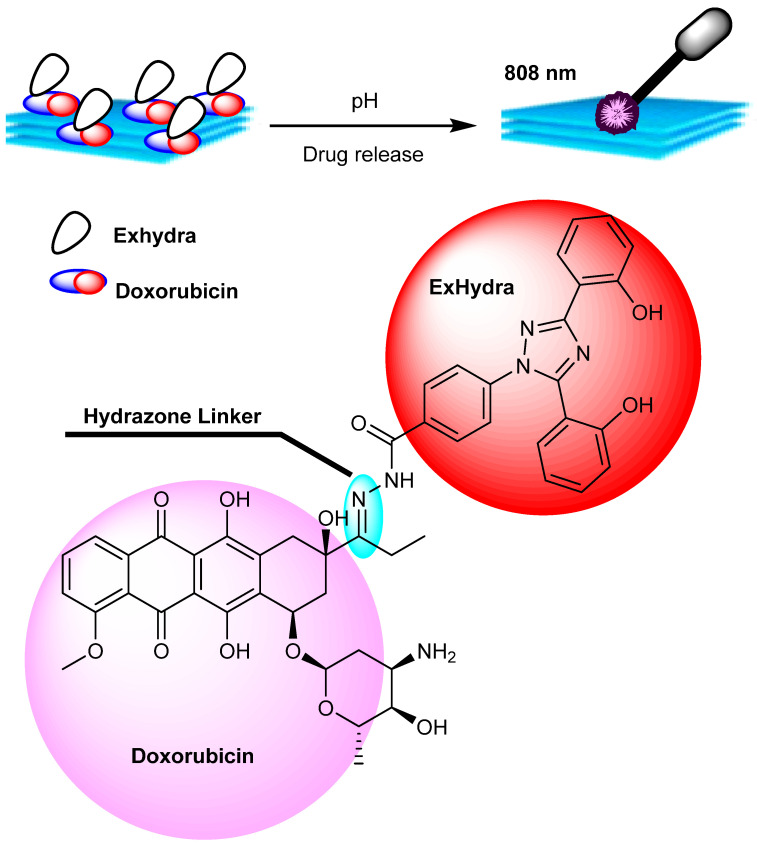
Synthesis of 2D-ultrathin MXene/DOXjade platforms for targeted cancer therapy [95].

**Figure 4:**
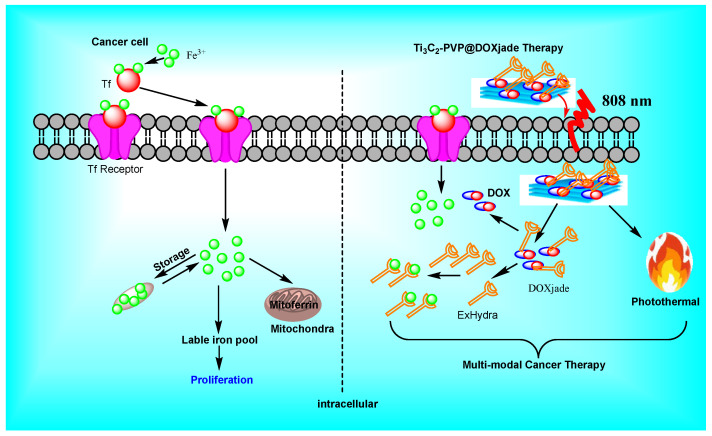
A plausible mechanism of 2D-ultrathin MXene/DOXjade platform for cancer therapy [95].

Two-dimensional ultrathin Ti_3_C_2_ MXene nanosheets were predicted to react with DOXjade in this setting. The ensuing construct, known as Ti_3_C_2_-PVP@DOXjade, was projected to have high photothermal efficiency [96]. Indeed, when exposed to NIR light at 808 nm inside the optical-therapeutic window (650–900 nm), Ti_3_C_2_ nanosheets included in Ti_3_C_2_-PVP@DOXjade demonstrated photothermal conversion efficiencies up to 40% and bestowed a good PTT performance [97,98]. Furthermore, Ti_3_C_2_-PVP@DOXjade flaunted a sensitive tumor pH-responsive iron chelation/PTT/chemotherapy anticancer impact. To remove the Al layer, Ti_3_AlC_2_ Max phase crystals were etched in an aqueous HF solution to generate Ti_3_C_2_ which was treated with an aqueous tetrapropylammonium hydroxide (TPAOH) solution and after agitation, the multilayer Ti_3_C_2_ nanosheets were formed. MXenes nanosheets and PVP (polyvinylpyrrolidone) were mixed in phosphate-buffered saline to afford Ti_3_C_2_-PVP; notably, the DOXjade was produced via the modification of DOX to offer hydrazones [99]. In phosphate-buffered saline (PBS) at pH = 7.4, DOXjade was loaded on Ti_3_C_2_-PVP under an argon atmosphere to afford DOXjade-loaded Ti_3_C_2_-PVP (Figure 3 and Figure 4).

### 2.4. Ti_3_C_2_ Nanosheet-Based Camouflaged Bionic Cascaded-Enzyme Nanoreactor

**Figure 5:**
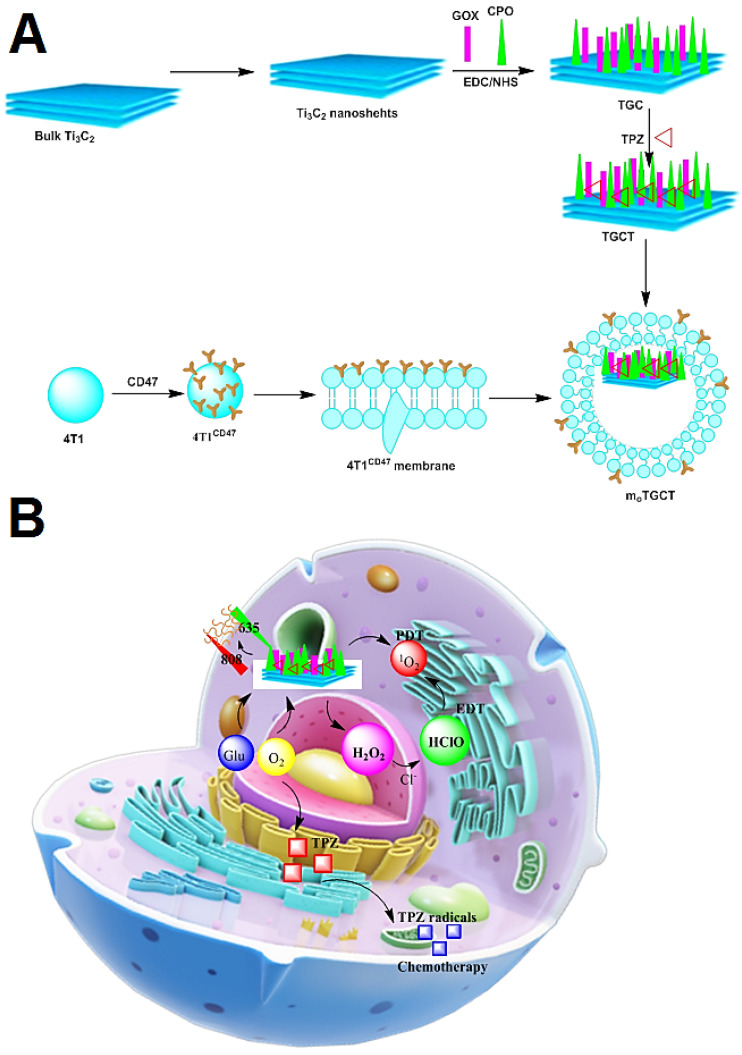
(**A**) Schematic diagram of Ti_3_C_2_ nanosheet-based camouflaged bionic cascaded-enzyme nanoreactor. (**B**) The possible mechanism of action for the designed MXene-based camouflaged bionic cascaded-enzyme nanoreactor (in vivo) [100].

The use of enzymes to generate ROS at tumor locations has emerged as a new technique for controlling intracellular redox states in anticancer therapy. The Ti_3_C_2_ nanosheet-based camouflaged bionic cascaded-enzyme nanoreactor has been applied for combined tumor enzyme dynamic treatment (EDT), phototherapy, and deoxygenation-activated chemotherapy [100]. The deoxygenation-activated drug tirapazamine (TPZ) was loaded onto Ti_3_C_2_ nanosheets, glucose oxidase (GOX), and chloroperoxidase (CPO) to furnish Ti_3_C_2_-GOX-CPO/TPZ (TGCT) which was implanted into nanosized cancer cell-derived membrane vesicles with high-expressed CD47 (meTGCT). The cascade reaction of GOX and CPO, into tumor cells, might create HClO for the effective EDT. Simultaneously, additional laser irradiation would speed up the enzymic-catalytic reaction rate and boost singlet oxygen production (^1^O_2_). A local hypoxic environment combined with EDT-induced oxygen depletion activates a deoxygenation-sensitive prodrug for treatment. Therefore, the synergistic therapeutic benefits of tumor phototherapy, EDT, and chemotherapy are increased in meTGCT. This cascaded-enzyme nanoreactor affords a good method to bring in anticancer treatment (Figure 5A) [100]. TGCT nanocascaded enzymes were created through the reaction of GOX and CPO onto Ti_3_C_2_ nanosheets with TPZ drug loading and the biomimetic alteration of CD47-overexpressed cancer cell membrane offer a bionic cascaded-enzyme nanoreactor (as meTGCT). MeTGCT was affected by tumor cells and the enzyme consumes glucose and oxygen in tumor areas to generate the lethal HClO. Under NIR laser irradiation, Ti_3_C_2_ may create both, heat, and ROS, where heat can speed up the enzyme-catalyzed reaction rate and ROS generation, exacerbating the hypoxic state in the target tumor environment (TME). Then, as a hypoxia-activated prodrug, TPZ is activated by reductase, causing DNA breakage and cell death, thus enhancing EDT and phototherapy effects. As a result, hypoxia-activated chemotherapy may achieve magnified synergistic effects in this camouflaged cascaded-enzyme nanoreactor, effectively inhibiting tumor development [100] (Figure 5B).

### 2.5. Few-Layered Nb_2_C (FNC) for Osteoclastogenesis Suppression Inhibited Inflammation and Osteoclastogenesis

**Figure 6:**
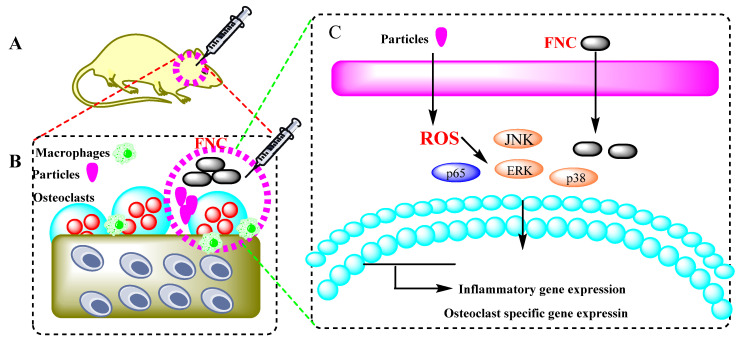
A schematic diagram of FNC inhibited inflammation and osteoclastogenesis. (**A**) Local injection of FNC at osteolytic sites. (**B**) Materials interaction between macrophages and osteoclasts influenced inflammation and osteoclastogenesis. (**C**) FNC was phagocytosed, and FNC exhibited the effect of ROS adsorption. Subsequently, the inflammatory and osteoclast-specific genes were down-regulated [101].

Implant-derived particulates, such as ultra-high molecular-weight polyethylene (UHMWPE), activate the immune system to phagocytose particles, resulting in the production of ROS [101]. ROS stimulates osteoclast genesis and causes macrophages to release cytokines, which aid in the progression of osteolysis. The few-layered Nb_2_C (FNC) is an antioxidant with the ability to reduce cytokine production and inhibit osteoclast genesis by ROS adsorption. Furthermore, in a mouse calvarial model, local injection of a few-layered Nb_2_C (FNC) reduces UHMWPE and induced osteolysis. As a result, it has been demonstrated that FNC could be beneficial in the treatment of osteolytic bone disease encouraged by excessive osteoclast genesis. In this process, the reaction of Nb_2_AlC, LiF, and HCl provided bulk Nb_2_C (BNC) which was treated with tetrapropylammonium hydroxide (TPAOH) to offer FNC. The ensuing product (few-layered Nb_2_C) was washed with DI water, centrifuged, and dried. It was demonstrated that FNC was effective in scavenging ROS for treating ROS-related diseases via FNC adsorption of ROS in the process of osteoclast genesis suppression in vitro. Furthermore, the findings displayed that osteolysis was condensed in vivo when UHMWPE was stimulated (Figure 6) [101].

### 2.6. Advanced Systems Based on Ti_3_C_2_ MXenes

**Figure 7:**
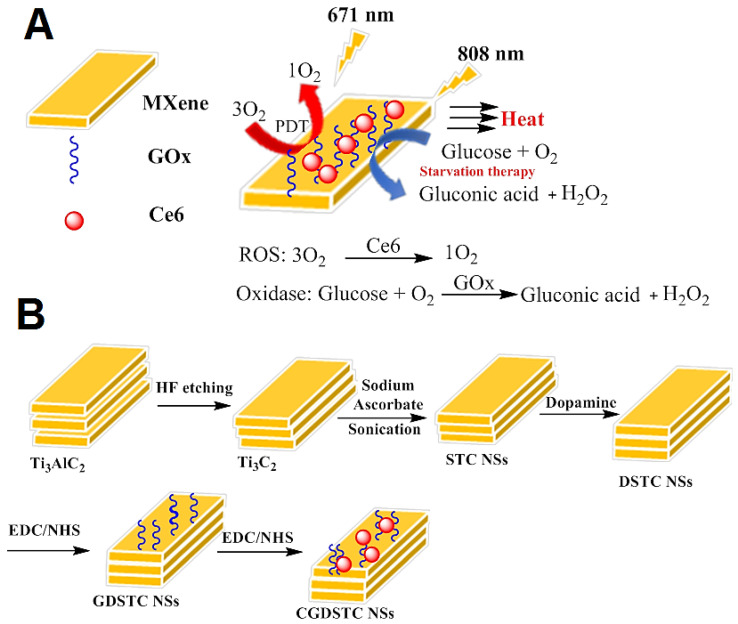
(**A**) Pictorial representation for the working mechanism of CGDSTC nanosheets. (**B**) The preparative process of CGDSTC nanosheets [33].

Titanium carbide (Ti_3_C_2_) MXenes were developed by surface modification in the presence of antioxidants (sodium ascorbate, SA, and dopamine, DA) to obtain (DA/SA@Ti_3_C_2_ = DSTC) [33]. Antioxidants improved the MXenes stability by trapping oxidants and the MXenes functionalization process with biotic molecules, therefore glucose oxidase (GOx) and photosensitizer (chlorin e6 = Ce6 PS) were attached to the DSTC surface through the carbodiimide link to provide Ce6/GOx@SDTC = CGDSTC). It was applied for glucose deprivation and photodynamic therapy for the co-killing of cancer cells. Increasing the solution temperature by laser irradiation boosted the enzymatic activity of CGDSTC nanosheets. These nanosheets exhibited cytocompatibility to HePG2 and HeLa cells; it was established that 90% of cells were killed by glucose and laser irradiation through the cooperative outcome between famine therapy and phototherapy. The possible mechanisms of synthesized CGDSTC nanosheets are illustrated in Figure 7A [33]. Glucose-rich environments provide gluconic acid in the tumor by GOx, subsequently sensitizing the tumor cells to photothermal properties by limiting the tumor glycolytic path. Photo irradiation with 808 and 671 nm lasers participates in tumor cell destruction by supporting Ti_3_C_2_- mediated PTT and Ce6-mediated PDT properties; higher temperature produces tumor oxygen levels alongside with GOx. Accordingly, synthesized CGDSTC nanosheets by starvation and phototherapy, remove the tumor cells more safely. The enzyme (GOx) and photosensitizer (Ce6) were sequentially conjugated onto the DSTC nanosheets via carbodiimide coupling between the carboxyl groups and amine groups of DSTC nanosheets to attain CGDSTC nanosheets with photodynamic and starvation properties (Figure 7B) [33].

Through the fluorescence imaging, MXene-based multifunctional nanosheets were developed to expand high cancer therapeutic efficacy via the cooperative effect. The stability of Ti_3_C_2_ MXene was improved by surface modification with the antioxidants such as sodium ascorbate (SA) and dopamine (DA) to offer DA/SA@Ti_3_C_2_ (abbreviated as DSTC).

### 2.7. MXene Quantum Dot/ZIF-Based Systems for Anticancer Drug Delivery

**Figure 8:**
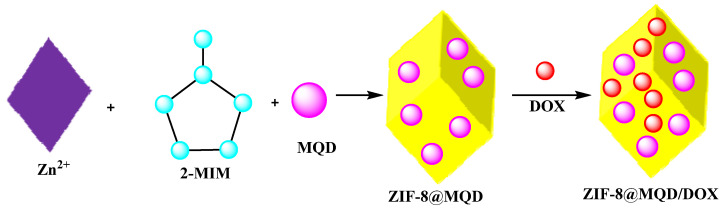
The preparative process of MXene quantum dot/ZIF-based systems for anticancer drug delivery [97].

Designing novel stimuli-responsive multifunctional structures for targeted drug delivery in cancer therapy has been studied [102,103]. For instance, zeolitic imidazolate framework-8 (ZIF-8) with a large and precise surface expanse, encapsulates DOX and MXene quantum dot (MQD) to furnish MQD@ZIF-8/DOX with high photothermal conversion efficacy and ROS generation ability to provide excellent photothermal therapy and photodynamic therapy effects. In addition, DOX was loaded into MQD@ZIF-8 nanoparticles which released the DOX at pH 5.6. Multifunctional MQD@ZIF-8 drug delivery is illustrated in Figure 8, where Zn^2+^ was immobilized on MQD as nucleation nodes. MQD@ZIF-8 composites were synthesized in situ via the rapid reaction between Zn^2+^ and 2-MIM molecules. The anticancer drug, DOX, was encapsulated in MQD@ZIF-8 where MQD served as PTA and PS in this system to realize combined PDT/PTT therapy, which rapidly converted NIR light energy to ablative heat and generated ROS under UV laser irradiation to kill cancer cells. Moreover, DOX release could be markedly increased under the tumor environment. The MQD@ZIF-8/DOX nanocarrier can therefore achieve the effect of combined chemotherapy and phototherapy during tumor treatment [102,103].

Multifunctional MQD@ZIF-8 drug delivery system was prepared by instantaneously capturing MQD with phototherapeutic potential and the chemotherapeutic drug DOX in a ZIF-8 environment. The MQD@ZIF-8 nanocarrier had a modest preparation and proficiently linked pH with NIR dual responsiveness to excite the drug release. Through the cell test, it was illustrated that MQD@ZIF-8 had a greater biocompatibility, and MQD@ZIF-8 could produce temperature and ROS around cells under 808 nm laser and UV irradiation for killing HeLa cells, individually. Thus, MQD@ZIF-8 could be considered as a suitable platform with superb tumor therapeutic potential.

### 2.8. Ionic Liquid-Exfoliated Ti_3_C_2_Tx MXene ((IL)-Ti_3_C_2_Tx MXene) Nanosheets in Cancer Treatment

**Figure 9:**
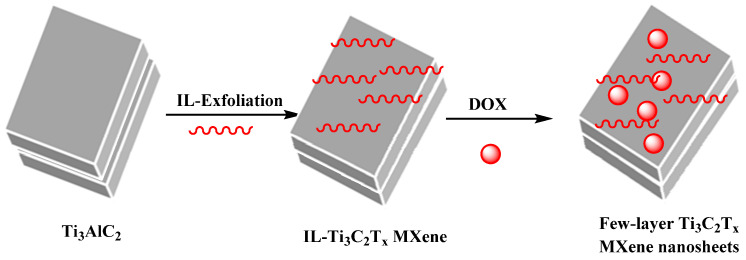
The preparation of few-layer Ti_3_C_2_Tx MXene nanosheets with high chemical stability using an IL-assisted exfoliating (red patterns) method for photoacoustic imaging-guided synergistic photothermal/chemotherapy of tumors [104].

Ti_3_C_2_Tx MXenes with potential activity in cancer treatment were produced through an ionic liquid (IL) stripping process; DOX was loaded into (IL)-Ti_3_C_2_Tx MXene to provide composites with pH-responsive behavior and photosensitivity with enhanced drug release upon 808 nm laser irradiation [104]. These nanocomposites exhibited high chemical stability and good biocompatibility. They could inhibit tumor growth using synergistic photothermal therapy and chemotherapy; photoacoustic imaging has great potential in cancer treatment (Figure 9) [104].

IL-supported exfoliation of the MXene phase could provide few-layer IL-Ti_3_C_2_T_x_ Under 808 nm laser irradiation, the IL-Ti_3_C_2_T_x_ MXene nanosheets exhibited high chemical stability and robust near-infrared absorption, with suitable photothermal conversion efficiency. The IL-Ti_3_C_2_T_x_ MXene nanosheets could be applied for targeted delivery of DOX with pH-/photosensitivity; the drug release was enhanced in an acidic situation under the laser irradiation (808 nm). These nanosheets displayed good photothermal effects on 4T1 cells under 808 nm (in vitro) to obtain the decent photoacoustic image. Furthermore, they exhibited appropriate antitumor properties (in vitro and in vivo). Thus, these MXene-based systems can be considered for photoacoustic imaging and synergistic photothermal/chemotherapy of cancer with high efficiency [104].

### 2.9. MXene@Au-Polyethylene Glycol Composites Drug Release

**Figure 10:**
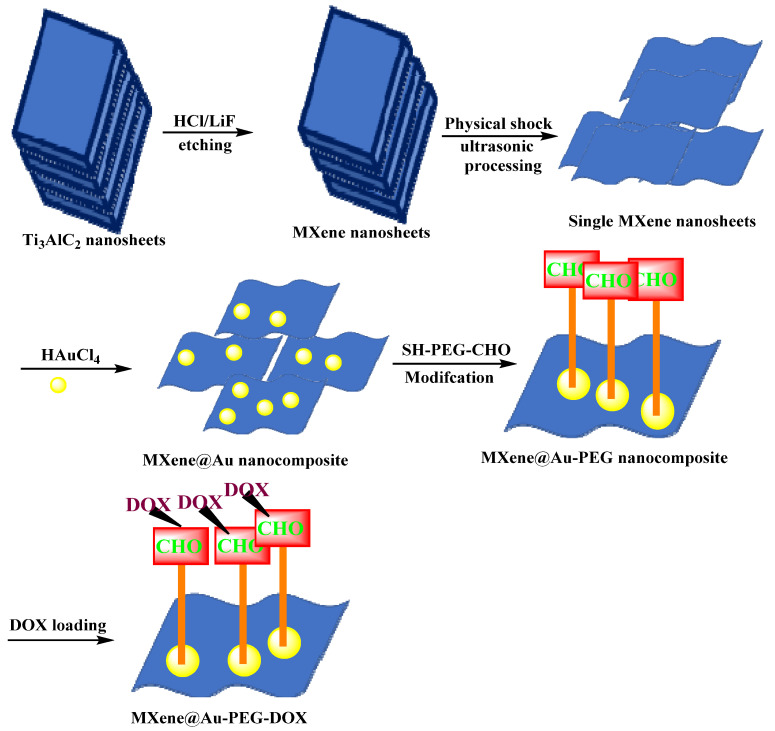
The preparative process and function of designed MXenes for cancer nanotherapy [106].

MXene@Au-polyethylene glycol composites have been developed as targeted drug delivery systems with a high capacity for loading chemotherapeutic agents (DOX) [105]; these nanosystems exhibited both NIR laser-triggered and pH-responsive drug release modes [106]. Owing to surface modification with thiol polyethylene glycol aldehyde chains (SH-PEG-CHO) connected to the MXene by Au nanoparticles, the system showed improved photothermal stability, biosafety, and histocompatibility during in vivo and in vitro tests. In addition, based on the good photothermal conversion capability of both Au nanomaterials and MXenes, these nanosystems exhibited synergistic photothermal ablation and chemotherapy for tumor/cancer therapy. Their passively targeted release properties also enhanced the cellular uptake of DOX at tumor sites, thus improving the efficiency of the drug. Future explorations ought to move towards designing surface-modified MXene-based drug delivery platforms with high drug-loading capacity, multiple drug release modes, and synergistic therapy, offering new opportunities for targeted cancer therapy and tumor ablation (Figure 10) [106].

A variety of MXene-based micro-and nano systems have been designed for targeted cancer therapy (Table 1).

## 3. Conclusions and Perspectives

MXenes, with their intrinsic physicochemical features and superb biocompatibility, have acquired a lot of interest in wide-ranging biological and biomedical applications. By managing preparative constraints, external features could be modified for cancer therapeutics and diagnostics. MXene-based micro- and nanosystems can be applied for targeted delivery of anticancer drugs/therapeutic agents, with the advantages of low toxicity and good biocompatibility. However, the improvement of biocompatibility, pharmacokinetics, and biodegradability of these structures ought to be focused comprehensively; discovering simple, cost-effective, and environmentally benign synthesis/functionalization approaches, optimized synthesis/reaction conditions, and efficient hybridization uniting biocompatible/biodegradable agents, are the key parameters in improving the properties and effectiveness of MXene-based systems. The verification of successful absorption of therapeutic chemicals from the circulation into the desired location is a key challenge in systemic disposition. The vascular endothelial cell, which acts as an obstacle between organs and blood, is one of the most important concerns. Endothelial cells’ cellular acceptance of manmade nanosystems is thus critical for their biological and therapeutic applications. MXene-based composites can be functionalized or modified utilizing appropriate functional groups/agents to enhance their loading capacity, biocompatibility, and bioavailability. This strategy can also be adopted to reduce undesirable/adverse side effects as well as probable immune system reactions. MXene-based materials for targeted drug delivery in cancer therapy would continue their fast progress made in less than half a decade in the future at a rapid pace. Hopefully, the analyses and outlook directions offered in this review can guide scientists and engineers to focus on the development of MXenes and their derivatives for targeted drug delivery and cancer therapy.

## Figures and Tables

**Table 1:** Different MXene-based systems for cancer therapy.

Formulations	Targeted Tissue/Organ	Advantages/Benefits	Biocompatibility and Toxicity	Ref.
Nb_2_C-Pt-DOX@M	Skin	Biomimetic plasmonic assembly completed the tumor treatment by irradiation of NIR-II, as a novel strategy to promote the nanozyme biocatalyst and plasmonic application in tumors.	Bioplasmonic assembly of biocatalyst	[94]
DOXjade-loaded Ti_3_C_2_-PVP	Skin	A pH-responsive dual-therapeutic compound based on federation and DOX, was created wherein photo-irradiation with Ti_3_C_2_-PVP@DOXjade displayed a pH-responsive iron chelation/PTT/chemotherapy anticancer activity.	Good biocompatibility and lower cytotoxicity	[95]
MeTGCT	Skin	Under NIR laser irradiation, Ti_3_C_2_ may create both heat and ROS, where heat can speed up the enzyme-catalyzed reaction rate and ROS generation, exacerbating the hypoxic state in the target TME.	Good biocompatibility	[100]
Few-layered Nb_2_C (FNC)	bone	Few-layered Nb_2_C (FNC) reduces UHMWPE and induced osteolysis.	Good biocompatibility	[101]
CGDSTC nanosheets	Skin	It was applied for glucose deprivation and photodynamic therapy for the cokilling of cancer cells	Higher stability and biologically safe under nonstimulus conditions	[33]
MXene quantum dot/ZIF-based systems	Skin	DOX and MXene quantum dot (MQD) to furnish MQD@ZIF-8/DOX with high photothermal conversion efficacy and ROS generation ability	Good biocompatibility, used as a drug delivery platform.	[97]
Few-layer Ti_3_C_2_Tx MXene nanosheets	Skin	photoacoustic imaging and synergistic photothermal/chemotherapy of cancer.	Good solubility, nontoxicity	[104]
MXene@Au-polyethylene glycol composites	Skin	Improved photothermal stability, biosafety, and histocompatibility during in vivo and vitro tests.	Good biocompatibility	[106]

## Data Availability

Not applicable.
